# Blood-inspired random bit generation using microfluidics system

**DOI:** 10.1038/s41598-024-58088-6

**Published:** 2024-03-29

**Authors:** Inkwon Yoon, Jong Hyeok Han, Byeong Uk Park, Hee-Jae Jeon

**Affiliations:** 1 Department of Mechanical and Biomedical Engineering, Kangwon National University, Chuncheon, 24341 Korea; 2 Department of Smart Health Science and Technology, Kangwon National University, Chuncheon, 24341 Korea; 3 Department of Advanced Mechanical Engineering, Kangwon National University, Chuncheon, 24341 Korea

**Keywords:** Whole blood, Laser speckle image, Random number generation, Decorrelation time, Biological techniques, Biotechnology

## Abstract

The development of random number generators (RNGs) using speckle patterns is pivotal for secure encryption key generation, drawing from the recent statistical properties identified in speckle-based imaging. Speckle-based RNG systems generate a sequence of random numbers through the unpredictable and reproducible nature of speckle patterns, ensuring a source of randomness that is independent of algorithms. However, to guarantee their effectiveness and reliability, these systems demand a meticulous and rigorous approach. In this study, we present a blood-inspired RNG system with a microfluidics device, designed to generate random numbers at a rate of 5.5 MHz and a high-speed of 1250 fps. This process is achieved by directing a laser beam through a volumetric scattering medium to procure speckle patterns. Additionally, designed microfluidic device requires only a minimal blood sample of 5 µl to capture these speckle patterns effectively. After implementing the two-pass tuple-output von Neumann debiasing algorithm to counteract statistical biases, we utilized the randomness statistical test suite from the National Institute of Standards and Technology for validation. The generated numbers successfully passed these tests, ensuring their randomness and unpredictability. Our blood-inspired RNG, utilizing whole blood, offers a pathway for affordable, high-output applications in fields like encryption, computer security, and data protection.

## Introduction

The evolution and continued development of Random Number Generator (RNG) systems remain a critical cornerstone for the future of cryptographic fields^1^. These RNG systems form the backbone of all cryptographic systems due to their role in generating keys that are not only difficult but nearly impossible to predict. This unpredictability ensures a high level of security, a necessary feature in today's digital world^2,3^. There are primarily two types of random number generators: algorithmic and physical. Algorithmic generators, also known as pseudorandom number generators (PRNGs)^4–6^, generate sequences of numbers using complex mathematical formulas. While they are convenient and efficient, PRNGs have a drawback as the generated pseudorandom numbers, even if they pass randomness tests, can be reverse-engineered and predicted. Physical (or true) random number generators, on the other hand, derive randomness from stochastic physical processes^7,8^. Although these processes are theoretically predictable with complete information, they are practically unpredictable due to limitations in time and computational resources^9,10^. RNGs of this nature play a vital role in various cybersecurity tasks, including key generation, digital signature creation, initialization vectors for cryptography, and generating salt values for secure storage, addressing the cybersecurity needs of interconnected systems beyond the IoT domain^11^.

One emerging area of interest is the use of optical physical unclonable functions for enhanced security. Research by Di Falco et al. has shown that chaotic systems^12^, facilitated by silicon chips, can be utilized for achieving a cryptography system with perfect secrecy^13–15^. However, it's essential to note that physically-based RNGs come with their own sets of challenges. For instance, while capturing random patterns is an innovative method, it suffers from limited bandwidth^16^. Another method, employing a light-emitting diode and a mobile phone camera, captures randomness from quantum fluctuations in light but is prohibitively complex to set up^17,18^. Hence, the landscape of RNG systems has been marked by a shift in recent years. In addition, conventional RNGs, which are artificially generated, have increasingly been found to contain inherent security vulnerabilities. These vulnerabilities have sparked a move towards more unpredictable, and inherently random, bio-inspired RNG systems. This shift acknowledges the fact that biological systems often contain levels of complexity and randomness that are difficult, if not impossible, to artificially replicate. Hence, recent RNG techniques have attempted to focus on the generation of RNGs using speckle patterns. The primary aim of these techniques is to achieve a balance of affordability and portability, both of which are vital factors for the widespread adoption and application of these systems^19,20^.

In the realm of speckle pattern generation, advancements in RNG and photonic physical unclonable functions often rely on intrinsic randomness in materials, such as the natural texture of paper^21^, as have speckle patterns created under coherent light. For instance, optical waveguides have demonstrated their ability to produce random numbers at Mbit/s rates with verified randomness^22^. Moreover, Fratalocchi et al. developed an all-optical physical unclonable function based on speckle patterns from aerogels, achieving secure key generation^12^. However, these techniques involve capturing static speckle patterns by passing a laser beam through a volumetric scattering medium. This approach, while innovative, poses challenges for cost-effectiveness and high-speed random number generation. Moreover, despite the enhanced security features offered by these RNGs, their application within the industrial field has been limited. The major hurdle lies in the high costs associated with sample fabrication and acquisition speed. As costs rise, the speed of acquisition generally improves; conversely, lower costs often result in decreased speeds, presenting a trade-off that complicates optimization. This issue creates a need for RNG systems that provide superior performance while also significantly reducing the costs of fabrication.

To address these challenges, we present an innovative RNG system that utilizes blood flow to generate speckle patterns via a microfluidic device. The distinct rheological properties of blood, enriched with a variety of cellular components, naturally enhance the complexity and entropy of the resulting speckle patterns, boosting the randomness of the generated numbers^23^. One of the standout features of our system is its modest sample requirement only 5 μl of blood is needed to capture speckle patterns at a speed of 1250 fps. This minimizes both costs and acquisition time, thereby increasing the system's accessibility. Our RNG system is the two-pass tuple-output von Neumann (2P-TO-VN) debiasing method, designed to ensure that the generated random bits are unbiased. This contributes to the overall unpredictability and robust security of the RNG while also increasing the number of generated bits. Furthermore, we confirmed a versatile key generation strategy, rigorously tested to meet the National Institute of Standards and Technology (NIST) standards for cryptographic applications^24^. This guarantees the generation of keys that are not only truly random but also entirely uncorrelated with one another, adding an additional layer of reliability.

## Results

### Speckle decorrelation time difference of random bits

**Figure 1:**
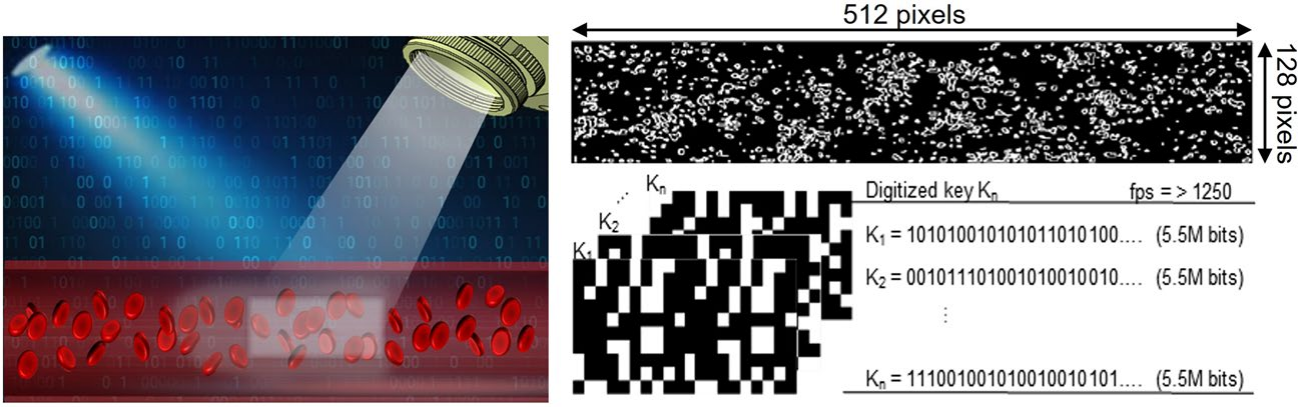
Schematic diagram to generate random numbers using blood flow. We employ blood flow within a microfluidic device to capture raw speckle patterns, which are then processed using 2P-TO-VN debiasing algorithm. This procedure corrects any biases in the raw speckle images, resulting in the generation of truly unpredictable random numbers.

**Figure 2:**
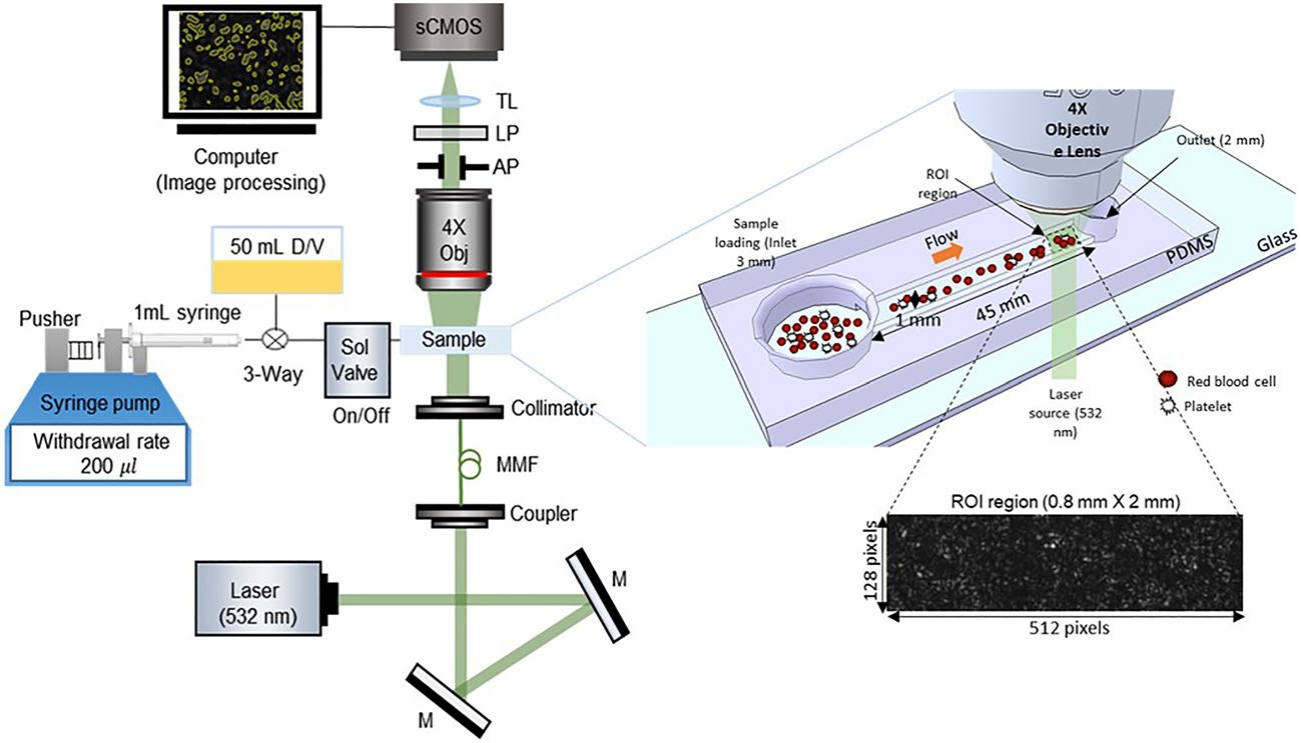
Schematic diagram to capture speckle pattern with whole blood. The green (λ = 532 nm) laser light passes through a microchannel, and the ROI region is captured by the objective lens of the microscope. The laser speckle images generated bio-inspired true random bit generation (TL: tube lens, LP: linear polarizer, AP: aperture, MMF: multi mode fiber, M: mirror, D/V: dead volume, ROI: region of interest, PDMS: polydimethylsiloxane).

**Figure 3:**
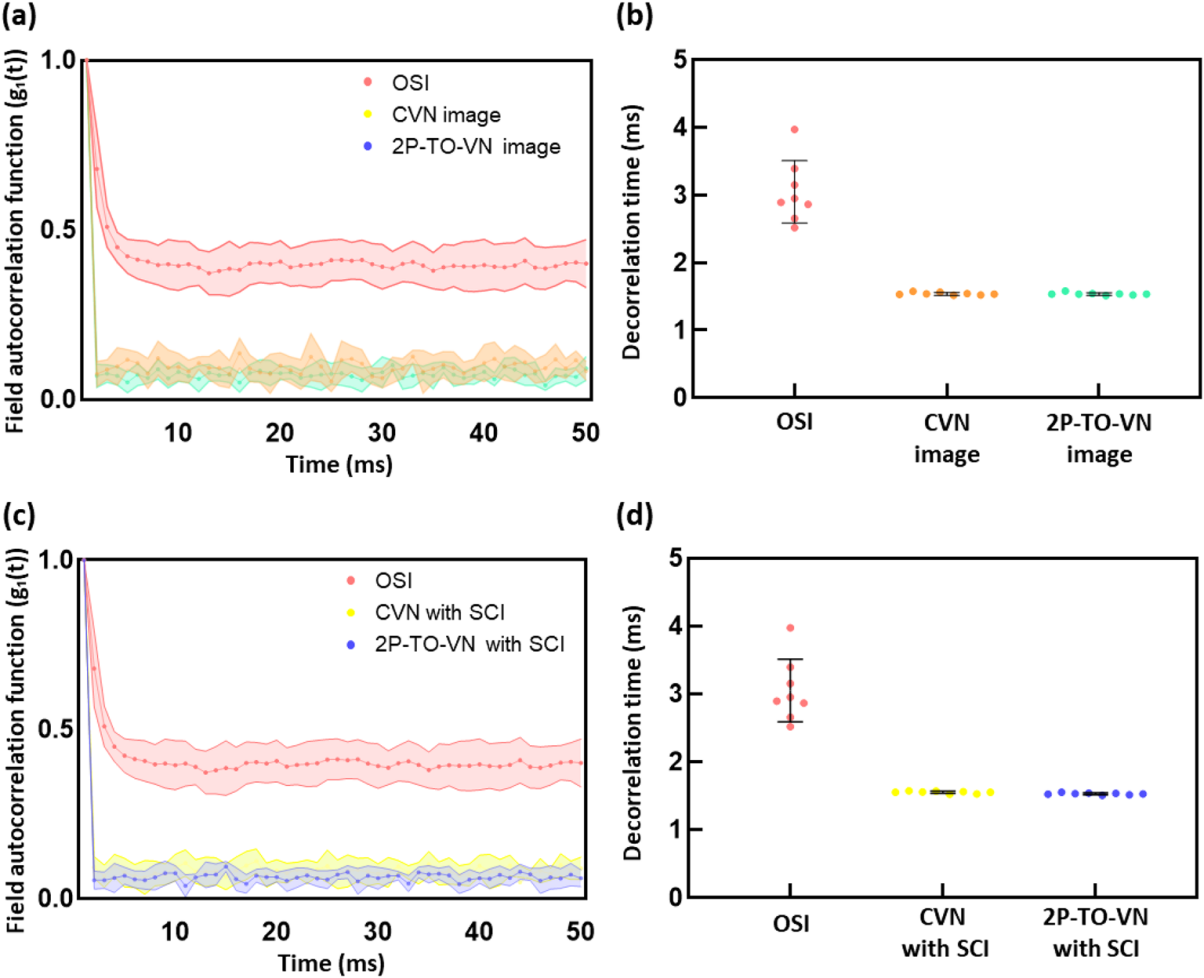
Speckle decorrelation time comparison between different algorithm types. (**a**) Speckle decorrelation curves for the original speckle image (OSI), classic von Neumann (CVN) image, and two-pass tuple-output von Neumann (2P-TO-VN) Image. (**b**) Comparison of decorrelation times for OSI, CVN Image, and 2P-TO-VN Image. (**c**) Speckle decorrelation curves for OSI, CVN with Scrambling Code Injection (SCI), and 2P-TO-VN with SCI. (**d**) Comparison of decorrelation Times for OSI, CVN with SCI, and 2P-TO-VN with SCI (This analysis was conducted by observing 7 different blood samples for each group). The correlation release time indicates the time it takes for the correlation between the initial image and subsequently captured images to decrease to 50%.

Figure 1 illustrates the method for capturing speckle images of blood flow, a technique that leverages the scattering properties of laser light to emulate the randomness found in blood circulation. Building on this concept, we designed a system to generate random numbers as depicted in Fig. 2, where decorrelation time is a key measure of estimated randomness. Figure 3a,b shows the autocorrelation curve across 56 original speckle images compared with true and scrambled random bits. A noticeable difference in decorrelation times emerged when comparing the original images to the derived random bits. The original speckle images had a mean decorrelation time of 3.05 s with a standard deviation of ± 0.43, while the series of post-processed random bits of the classic von Neumann (CVN) image, CVN with scrambling, 2P-TO-VN, and 2P-TO-VN with scrambling image demonstrated remarkably consistent means and standard deviations: 1.542 ± 0.019, 1.538 ± 0.018, 1.551 ± 0.017, and 1.529 ± 0.013, respectively in Fig. 3c,d. These closely clustered values indicate a low to non-existent correlation between the 56 varied images, underscoring the effective randomness introduced by our processing technique.

### Comparison of bit uniformity and correlation difference

**Figure 4:**
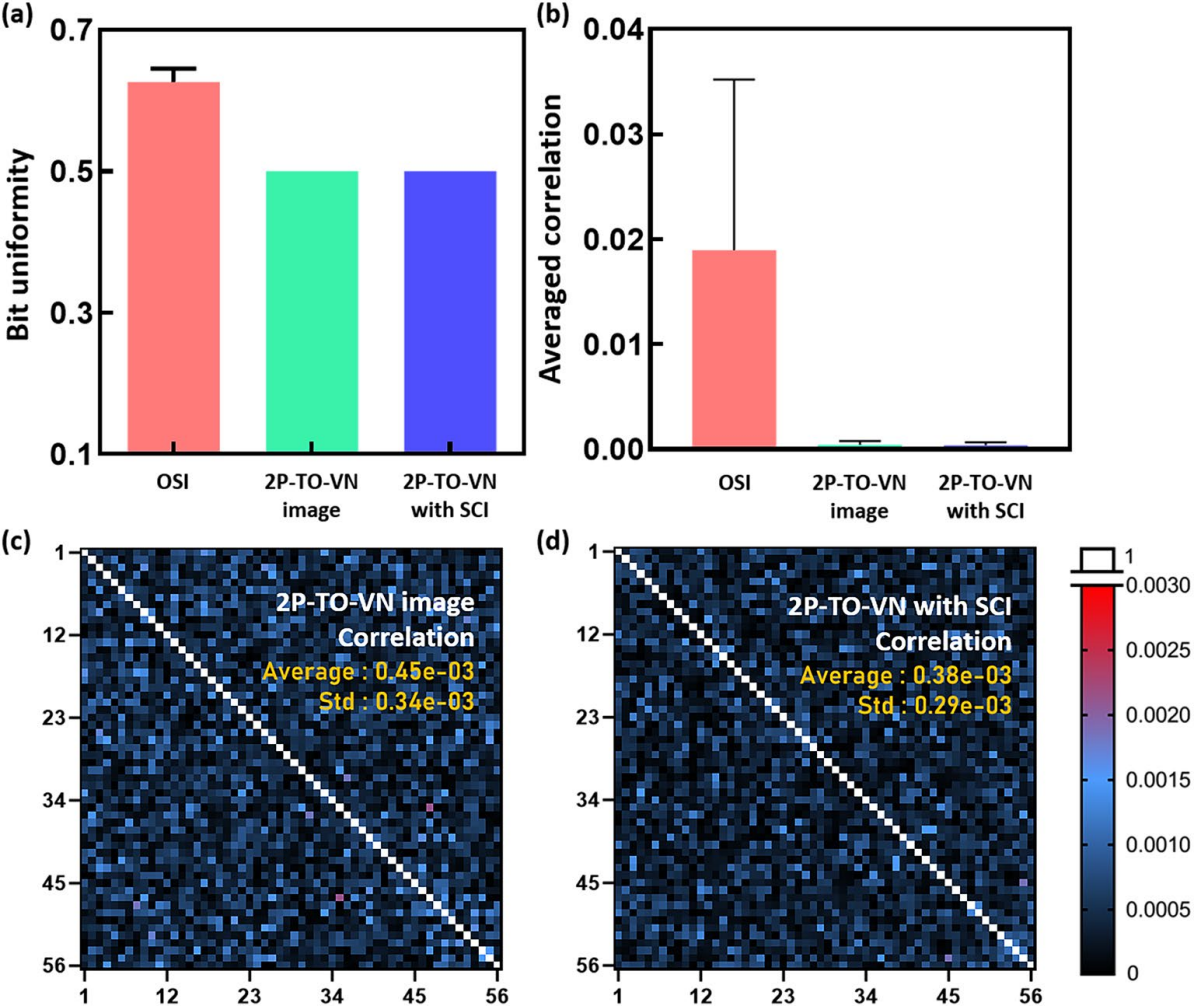
Comparison of characterizations of random number matrices from RNGs. (**a**) Bit uniformity for OSI, 2P-TO-VN Image, and 2P-TO-VN with SCI. OSI before removing the bias had an average bit uniformity value of 0.626, indicating that the bits were biased. In contrast, 2P-TO-VN and 2P-TO-VN with SCI after removing the bias are both 0.5, demonstrating an unbiased distribution between 0 and 1 states. (**b**) Comparison of Average Correlation Values for 56 Different OSI, 2P-TO-VN Image, and 2P-TO-VN with SCI. The average correlation values with standard deviation for each group are (0.18 ± 0.16) × 10^–2^, (0.45 ± 0.34) × 10^–3^, and (0.38 ± 0.29) × 10^–3^, respectively. (**c**) Correlation heatmap for 56 different 2P-TO-VN Images. (**d**) Correlation Heatmap for 56 Different 2P-TO-VN with SCI Images. In (**c**) and (**d**), correlation values outside the diagonal region approaching 0 indicate reduced correlation.

To address potential biases in our data, we employed the von Neumann debiasing algorithm and examined the bit uniformity of original speckle images, which averaged 0.626, reflecting a skew. Post-application of the 2P-TO-VN debiasing algorithm, we observed an exemplary bit uniformity of 0.500 for both sets of data processed with 2P-TO-VN alone and combined with SCI, as shown in Fig. 4a, indicating an ideal balance of '1' and '0' bits. Further scrutiny using correlation analysis on 56 original and processed speckle images, as shown in Fig. 4b-d, revealed that the processed datasets achieved correlation values significantly lower than those of the original images by approximately 2,270 times. Moreover, when comparing the two processed datasets, the 2P-TO-VN with SCI variant demonstrated an 18.34% reduction in correlation value compared to the 2P-TO-VN images alone, suggesting that SCI processing further attenuates correlation. Specific correlation metrics for the original speckle images recorded an average of 0.01896 with a standard deviation of 0.016264. The processed data showed marked improvement, with 2P-TO-VN images having correlation values of 0.000453 and 0.000343, and 2P-TO-VN with SCI producing even lower correlations of 0.000383 and 0.000296. These findings robustly underscore the effectiveness of the 2P-TO-VN algorithm in achieving bit uniformity and minimizing correlation, thereby validating the statistical reliability of our random number generator based on blood flow speckle dynamics.

### Comparison of random bit generation rate

**Figure 5:**
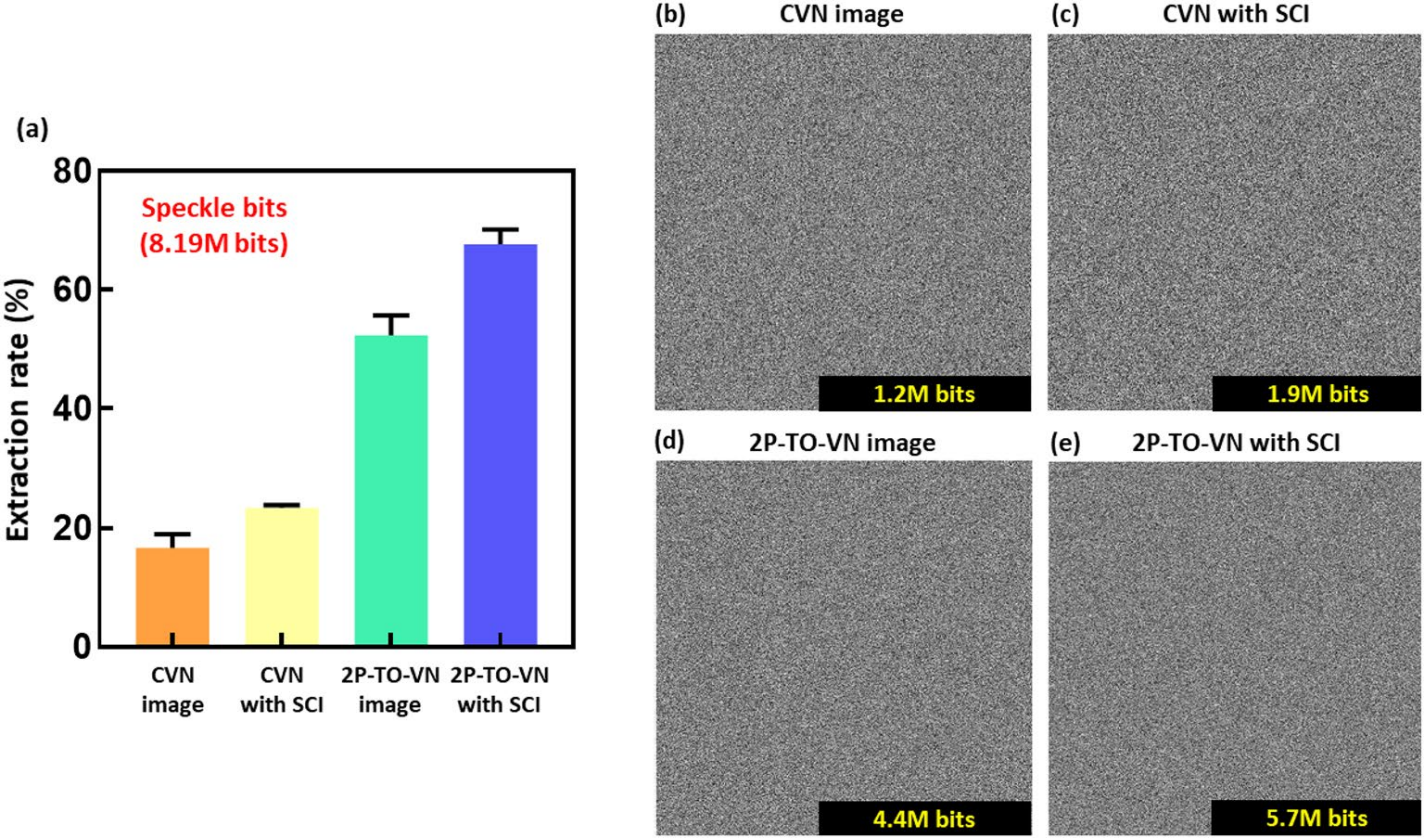
The results of bit generation rate and representative binary bitmaps of cryptographic keys. (**a**) Graph of bit generation rate generated from speckle bits. It shows the bit generation rates for CVN image, CVN with SCI, 2P-TO-VN image, and 2P-TO-VN with SCI obtained from 56 different random bits. The speckle bits consist of 8.1 million bits. The average and standard deviation of each image were 16.65 ± 2.27, 23.37 ± 0.49, 52.35 ± 3.34, and 67.70 ± 2.44, respectively. (**b-e**) Representative binary bitmaps of bitstreams generated by different algorithms (CVN image, CVN with SCI, 2P-TO-VN image, 2P-TO-VN with SCI). These binary bitstreams have been tested for unpredictability through NIST randomness tests.

The ability to extract a large number of bits is crucial, reflecting the potential to generate high-quality random sequences for applications across technology and science^25,26^. Notably, the initial analysis of speckle images consistently generated an output of 8.19 million bits on Fig. S1. Figure 5 clarifies this by illustrating the relationship between the bit generation rate and the volume of bits successfully retrieved. When processed via the CVN algorithm, the bits derived from both CVN images and those with SCI display average generation rates of 16.65 ± 2.27% and 23.37 ± 0.48%, respectively. By contrast, employing the 2P-TO-VN algorithm, which includes processing of the 2P-TO-VN image and 2P-TO-VN with SCI, yields higher average generation rates of 52.35 ± 3.34% and a more impressive 67.70 ± 2.43% for the 2P-TO-VN with SCI. These statistics underscore the benefit of the scrambling technique in enhancing the extractable bit quantity and validate the 2P-TO-VN algorithm's superiority over CVN in boosting bit generation efficiency^27,28^.

### The result of NIST statistical test

The purpose of the NIST tests is to validate the security and unpredictability of digital encryption methods^25,29^. Table 1 shows chi-square (X^2^) test data, reflecting *p* values and acceptance ratios for analyzing random bit patterns. We segmented 56 unique bit patterns into 60 sequences for detailed statistical review. According to the table, all the bit patterns except those from the original speckle images have passed the stringent criteria of the seven NIST randomness assessments. For more specific information about the testing parameters and the rigorous standards of the NIST suite, refer to the supplementary Table S1. The original speckle image failed the NIST Statistical Randomness Tests for Binary Sequences (Table S2). However, the result of the CVN image by NIST Statistical Randomness Tests for Binary Sequences (Table S3), and the result of CVN with SCI by NIST Statistical Randomness Tests for Binary Sequences (Table S4), 56 2P-TO-VN image and 2P-TO-VN with SCI, successfully passed (Table 1a,b). These results confirm the statistical randomness of the processed bit sequences, proving their efficacy as secure, non-deterministic encryption keys for cryptographic applications.

**Table 1:** NIST statistical randomness tests for binary sequences generated from blood. Summary of NIST statistical randomness test results collected from (a) 56 2P-TO-VN image and (b) 2P-TO-VN with SCI.

NIST statistical test^a^	*p* value	Proportion	Result^b^
(a)			
Frequency	0.000000	60/60	Pass
BlockFrequency	0.049821	60/60	Pass
CumulativeSums	0.000000	60/60	Pass
	0.000000	60/60	Pass
Runs	0.498475	59/60	Pass
LongestRun	0.505842	59/60	Pass
ApproximateEntropy	0.341105	58/60	Pass
Serial	0.466500	59/60	Pass
	0.449781	59/60	Pass
(b)			
Frequency	0.000000	60/60	Pass
BlockFrequency	0.000000	60/60	Pass
CumulativeSums	0.000000	60/60	Pass
	0.000000	60/60	Pass
Runs	0.463253	60/60	Pass
LongestRun	0.462783	59/60	Pass
ApproximateEntropy	0.442474	59/60	Pass
Serial	0.439529	59/60	Pass
	0.497110	59/60	Pass

^a^The NIST statistical tests involve using a sequence of 4.8 M bits obtained from 56 different random bits. This bit sequence is then divided into 60 sequences, each consisting of 80,000 bits. The goodness-of-fit of the *p* value distribution is compared to an expected distribution using the χ^2^ distribution. The bit sequence is considered to be random only if the resulting *p* value is greater than or equal to 0.0001.

^b^A test is considered to have passed if the pass rate exceeds the minimum rate of greater than 56 out of 60 tests.

## Discussions

We have demonstrated a means to extract large-scale encryption keys inspired by biological processes. Using a small amount of blood through a microfluidic chip, we can obtain non-reproducible speckle images. The system described above utilizes blood flow, making it impossible to retrieve the same encryption key even when produced and experimented under identical conditions. This underscores the unpredictable bio-inspired RNG system as a complement to the security vulnerabilities of existing artificially generated RNGs. We captured the region of interest (ROI) using a small amount of blood (5 µl) and recorded it at a speed of 1250 fps using an sCMOS camera. To emphasize its non-periodic nature, we demonstrated that it is a high-speed RNG system based on the results of speckle decorrelation time and correlation average. Furthermore, this point indicates that as the frame rate increases, the amount of data also increases, which can be influenced by the camera’s performance. We transformed speckle images into random bits that can be used as large-scale encryption keys using the von Neumann extractor. Through the 2P-TO-VN debiasing algorithm, which is better than CVN, we can generate encryption keys with low compression rates and remove bias while maintaining practical data sizes. This was validated through bit uniformity and data volume, and it was demonstrated that the statistical properties were improved through the NIST statistical test.

The demand for highly secure methods to safeguard data, infrastructure, medical, and of financial transactions is paramount as society becomes more interconnected, particularly in the rapidly expanding Internet of Things (IoT) market^30^. In practical terms, the blood-inspired RNG system we've developed offers a promising avenue for real-world applications, particularly in areas requiring high security and data integrity. For instance, in the financial sector, this system could revolutionize the way sensitive transactions are encrypted, providing an additional layer of security against cyber threats. In healthcare, patient data could be protected with encryption keys generated through this system, ensuring confidentiality and privacy. Additionally, in the realm of cybersecurity, the unique and unpredictable nature of the keys generated by our system could significantly bolster defenses against hacking and unauthorized data access. The potential of this technology extends even further, including secure communications in defense and intelligence operations, where the integrity and confidentiality of information are paramount. By offering a high-speed, cost-effective, and highly secure RNG solution, our blood-inspired system is poised to redefine security protocols across these critical sectors.

In conclusion, we have showcased a new approach to RNG systems inspired by biological processes. This development suggests new possibilities for RNG applications, with the presented blood-inspired RNG technology affirmatively demonstrating the generation of true random number. The use of blood in creating RNG systems offers unique advantages, showcasing its role in enhancing randomness and security in various applications. Our innovative RNG system offers a two-fold advantage. First, it reduces the costs associated with RNG generation, making it a cost-effective solution. Second, it boosts output, enhancing the performance of the system. The potential applications of our blood flow-inspired RNG are extensive. It is poised to make a significant impact across various fields, including cryptography, computer security, and data encryption. Our study heralds a new frontier in RNG systems. By marrying affordability with high output, it opens up new possibilities and sets the stage for future advancements in the field of RNG systems.

## Methods

### Bio-inspired random number generation system

In our setup, a 50-mW green laser with a wavelength of 532 nm (PSU-III-LCD, Changchun New Industries Optoelectronics Tech Co., Ltd., China) directs its beam through a microfluidic chip. Blood flow within the microchannel is brought into focus under a 4X objective lens (Plan N 4X, NA 0.1, Olympus), and a speckle image is then recorded using a CMOS camera (Neo 5.5 sCMOS, Andor Technology Ltd., Belfast, UK. This CMOS camera operates with an exposure time of 0.8 ms and a high frame rate of 1250 frames per second, seamlessly capturing speckle images at a resolution of 128 × 512 pixels for subsequent data generation. To ensure a consistent starting point, data was extracted from 1000 frames, forming a total of 7 sets of experimental data. To preprocess the experimental data, we divided it into 8 partitions, each consisting of 32 × 256pixels, resulting in a total of 56 sets. Through this process, our RNG system, utilizing blood, has been confirmed to generate true random numbers at a rapid speed of 5.5 MHz. To enhance the image contrast, a linear polarizer positioned at a 90-degree angle, an aperture with a 5 mm diameter, and a tube lens with a 180 mm focal length are positioned in front of the camera. The schematic of the entire system is presented in Fig. 2.

### Blood sample preparation

Study has approved by the Laboratory Animal Resource Center of the Gwangju Institute of Science and Technology (LARC GIST), as detailed on their website [https://larc.gist.ac.kr/]. These procedures were rigorously followed and formally approved under protocol GIST-2019–015 and all methods are reported in accordance with ARRIVE guidelines for reporting experiments. We collected blood samples from male Sprague Dawley rats aged 12–13 weeks, weighing between 250 and 280 g, by tail vein phlebotomy. For this, we administered 1 ml of blood through a 23G needle while the animals were anesthetized with isoflurane. We conducted these blood collections on a group of 11 rats, ensuring a rest period of at least two weeks between each procedure. The samples were immediately stored in citrate tubes (catalog #363083, 9NC 0.109 M Buffered Trisodium Citrate, BD Vacutainer, USA) for subsequent experiments.

### Fabrication of microfluidic device and system operation

To construct the microfluidics channel as shown in Fig. S2, we applied soft photolithography to create channels with precise dimensions: 45 mm in length, 45 µm in height, and 1 mm in width. We made a PDMS slab by a standard process of mixing, degassing, and curing. This slab, composed of PDMS Sylgard 184 A/B (Dow Corning, South Korea), was then bonded to a cover glass via oxygen plasma treatment.

Our microfluidic system, detailed in Fig. 2b, comprised a vacuum generator incorporating a syringe pump in withdrawal mode, a solenoid valve, a 3-way valve, and a 50 ml syringe to account for dead volume. A syringe pump controlled the sample draw at a constant volume of 200 µL in fluctuation withdrawal mode, with a solenoid valve regulating the flow. During the experiment, we intentionally designed it with a large dead volume to stabilize pressure fluctuations. At the microchannel's outlet, we positioned a 1 mm diameter reservoir and observed it with an sCMOS camera. Before beginning our experiments, we allowed the laser a 5-min warm-up period to ensure stability. The monitoring process started when the blood sample was introduced at the inlet and flowed into the microchannel, reaching the designated ROI measuring 0.8 mm by 3.3 mm. Once in position, the solenoid valve was opened, and the camera began capturing images for analysis as the blood entered the ROI.

### Image processing to generate binary keys with a von Neumann extractor

**Figure 6:**
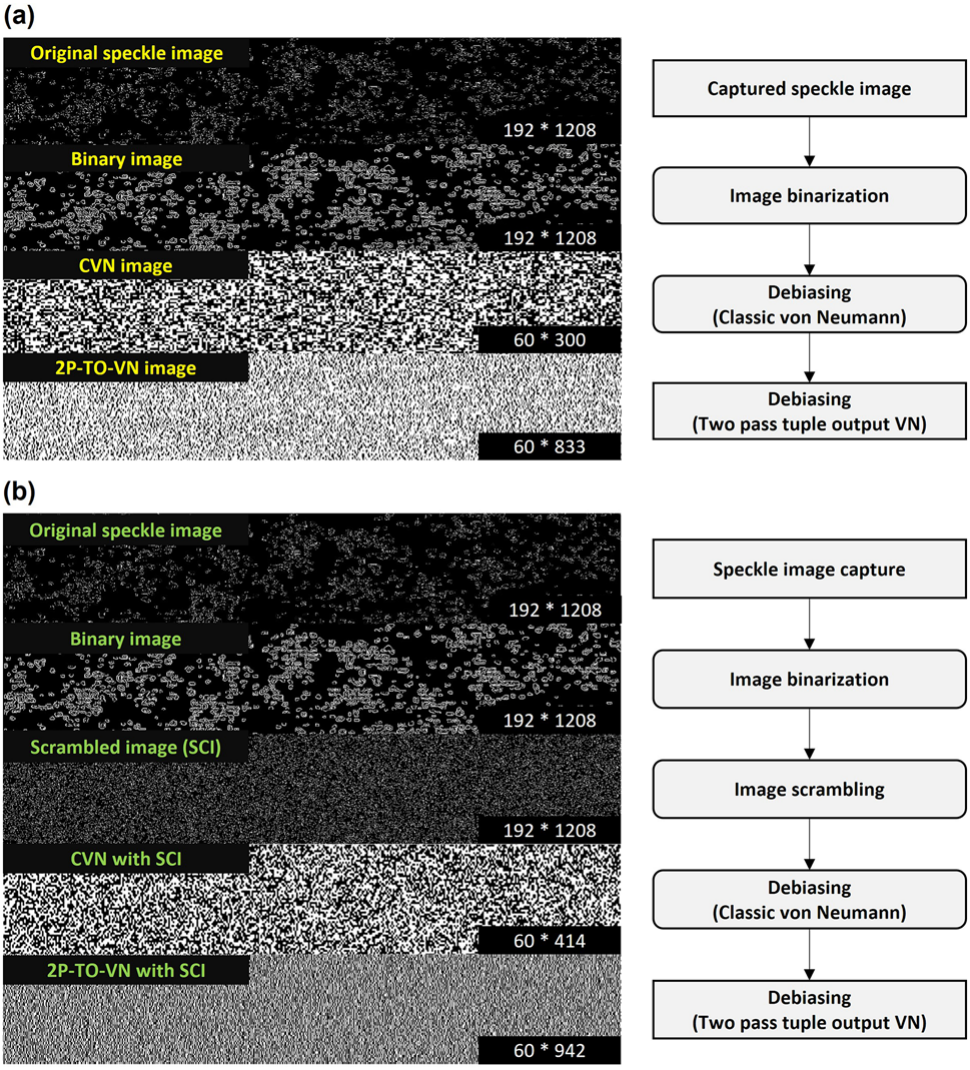
Flowchart for unpredictable random number generation using the speckle images. The speckle pattern produced unpredictable random bits consisting of 0 and 1 bits. To improve the high-output and performance, we incorporated additional randomness using image scrambling and 2P-TO-VN debiasing algorithm. (**a**) Binary image generation process using von Neumann debiasing algorithm (**b**) Binary image generation process using image scrambling and von Neumann debiasing algorithm. Total Pixels of 2P-TO-VN image and 2P-TO-VN with SCI is 49,980 (60 × 833) pixels and 56,520 (60 × 942) pixels, respectively. It shows that it is affected by scrambling.

We have devised an algorithm for processing speckle pattern images obtained from blood flow to generate encryption keys. The algorithm converts the original speckle images into binary sequences through a binarization process, which thresholds pixel values against the mean, categorizing them as '0' or '1'. Prior to applying our Two-Pass Tuple-Output von Neumann (2P-TO-VN) algorithm, we enhance the rate of bit generation by scrambling the image. This methodology was assessed both with and without image scrambling in Fig. 6a and 6b to determine its efficacy. To mitigate biases and correlations inherent in laser-produced speckle patterns and varying lighting conditions, we employ the following steps in our 2P-TO-VN debiasing algorithm generation:

1. Outputs of ‘00’ or ‘11’ are deleted.
2. If the output is ‘01’ or ‘10’ only the first bit, such as ‘0’ of ‘01’ or ‘1’ of ‘10,’ is retained.
3. In the second pass, bits discarded in the first pass are regrouped into quads, and the front and rear halves of each quad are compared.
4. Discarded bits with different front and rear halves, such as ‘0011’ or ‘1100,’ are preserved

While the CVN debiasing method, known as 1 Pass tuple output von Neumann (1P-TO-VN), greatly reduces bit count due to its stringent compression, it has been noted that 2P-TO-VN offers a balance between bit retention and debiasing. By re-evaluating initially discarded bits, the 2P-TO-VN algorithm generates a more feasible volume of data for practical applications, as demonstrated in Fig. S3, making it a superior alternative to CVN for generating robust and usable encryption keys.

### NIST statistical randomness tests for random bits generated from speckle images

To determine the quality of randomness of our random bits, we conducted a series of statistical tests using the National Institute of Standards and Technology Statistical Test Suite^31^. The NIST test suite is designed to quantitatively evaluate the randomness of binary sequences. The NIST test suite comprises 15 distinct tests, each designed to quantitatively measure different aspects of randomness in binary sequences. The tests include assessing frequency, block frequency, runs, longest runs of ones (LRO), serial, approximate entropy, cumulative sums (Cusums). Our evaluation involved aggregating binary sequences from 56 different random bits, ensuring an adequate stream length for seven of the statistical tests.

### Speckle decorrelation time measurement

Laser speckle is interference of light after multiple scattering from an optically turbid medium such as whole blood^32,33^. This laser speckle pattern changes over time as the sample conditions are changed. The speckle pattern data can be collected for time *t* with the interval of τ. To calculate the electrical field autocorrelation, we used the electrical field autocorrelation function ${g}_{1}\left(\tau \right)$. The ${g}_{1}\left(\tau \right)$ is defined as,

1 $${g}_{1}\left(\tau \right)={\int }_{0}^{\infty }P\left(s\right)exp\left[\left(-\frac{2\tau }{{\tau }_{o}}\right)\frac{s}{{l}^{*}}\right]$$

where *P(s)* is the path length distribution in the sample*, s* is the path length, τ is the delay time, l* is the transport mean-free path, and τ_o_ is the characteristic decay time of the medium. At zero, the autocorrelation is expected to be in a value of 1, which means there are no variation during at this time^34^. However, as the time lags increases, those values should drop to close to zero, in which time the signal is no longer correlated as compared first time images. For confirmation of the bio-inspired true random bit generation, we measured decorrelation time between the two consecutive images from the time series of speckle pattern images at the point of 50%, as the blood moves through the microchannel.

### Data analysis (bit uniformity, average correlation)

To evaluate the performance of random bits, we examine the digitized keys. Bit uniformity assesses how balanced the distribution of ‘0’ and ‘1’ bits is within the random bits. Specifically, it estimates how uniform the ratio of ‘0’s to ‘1’s is.

2 $$Bit\; uniformity=\frac{1}{s}\sum_{l=1}^{s}{K}_{l}$$

The uniformity of random bit sequences is often assessed by determining the Hamming weight, which is the count of '1' bits in a binary string of length 's'. For a set of random bits, the desired uniformity is achieved when the Hamming weight approaches 0.5, reflecting an equal distribution of '0' and '1' bits, as illustrated in Figs. 4a and S4a. Furthermore, we investigate the correlation between images using a correlation matrix. To ensure a fair comparison, random bits obtained through the von Neumann debiasing algorithm were quantified and matched to create uniform bit matrices. (e.g., original speckle image: 7.8 M bits, CVN image: 1.02 M bits, CVN with SCI: 1.8 M bits, 2P-TO-VN image: 3.3 M bits, 2P-TO-VN with SCI: 4.8 M bits).

3 $$r=\frac{\sum_{i=1}^{n}({x}_{i}-\overline{x })({y}_{i}-\overline{y })}{\sqrt{\sum_{i=1}^{n}{({x}_{i}-\overline{x })}^{2}}\sqrt{\sum_{i=1}^{n}{({y}_{i}-\overline{y })}^{2}}}$$

We employ the Pearson correlation coefficient formula to quantify the correlation^35^. In the formula, x̅ and y̅ represent the means of the x and y values. A Pearson correlation coefficient, denoted as r, close to 0 indicates that there is no significant correlation between the two sets of random bits^35^ in Fig. 4b,d, S4b, and S5.

### Supplementary Information

Supplementary Information.

## Data Availability

Correspondence and requests for materials should be addressed to H.J.
