# Supplementary material for: Blood-inspired random bit generation using microfluidics system
Source: Sci Rep. 2024 Mar 29;14:7474. doi: 10.1038/s41598-024-58088-6 (PMC10980712; doi:10.1038/s41598-024-58088-6)
Supplement: Supplementary file 1 — Supplementary Information. [file 41598_2024_58088_MOESM1_ESM.docx]

**Blood-inspired random bit generation using microfluidics system**

inkwon Yoon^1^, Jong Hyeok Han^1^, Byeong Uk Park^1^, Hee-Jae Jeon^1,2,3*^

^1^Department of Mechanical and Biomedical Engineering, Kangwon National University, Chuncheon 24341, Korea

^2^Department of smart Health Science and Technology, Kangwon National University, Chuncheon 24341, Korea

^3^Department of Advanced Mechanical Engineering, Kangwon National University, Chuncheon 24341, Korea

*Correspondence and requests for materials should be addressed H-J. Jeon (jeon22@kangwon.ac.kr).

**Supplementary Figures**

**
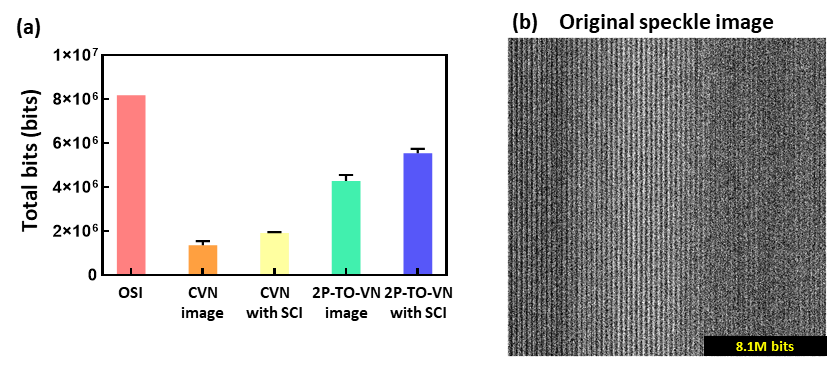
**

**Figure S1. The result of total bits and representative binary bitmap of cryptographic keys. (a)** The result of total bits, **(b)** Binary bitmap of original speckle image.

**
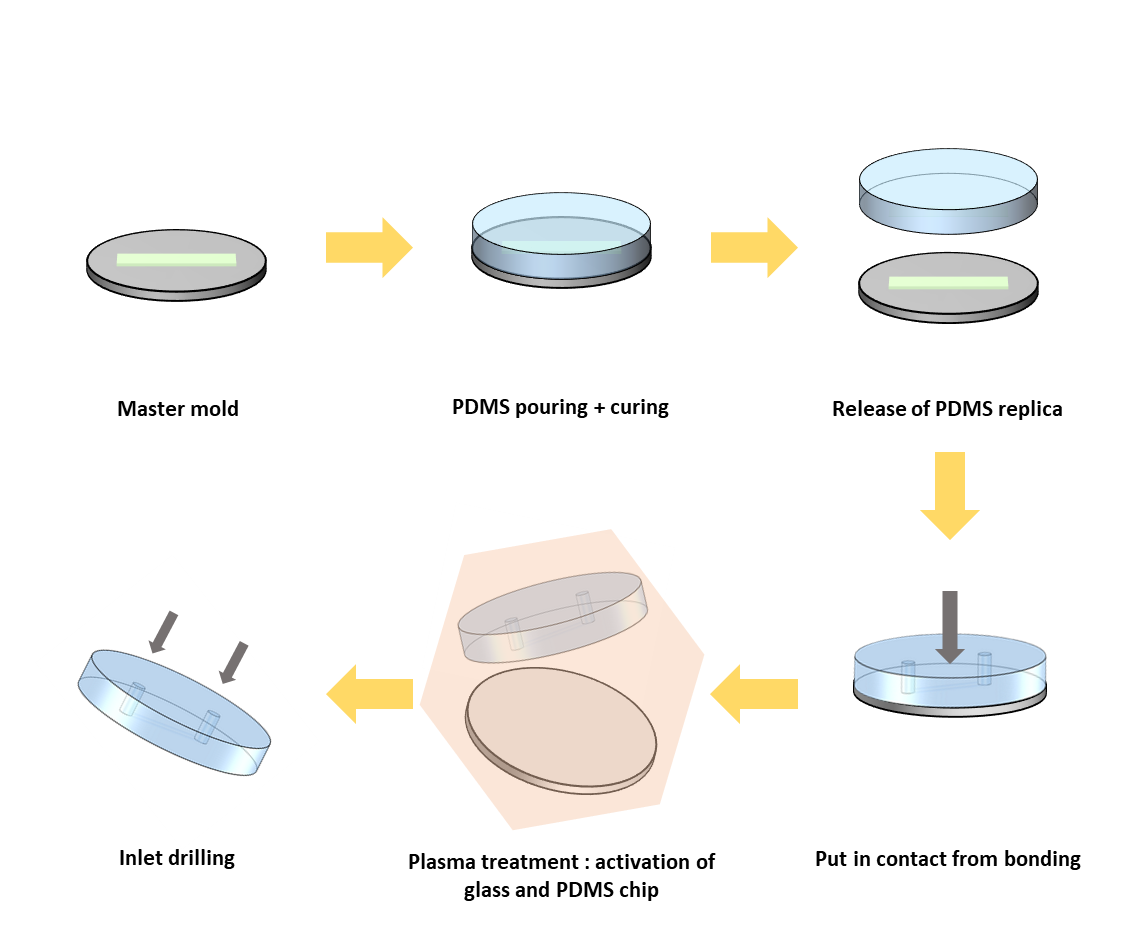
**

**Figure S2. Representation of the entire microfabrication sequence for a microfluidic chip.**

**
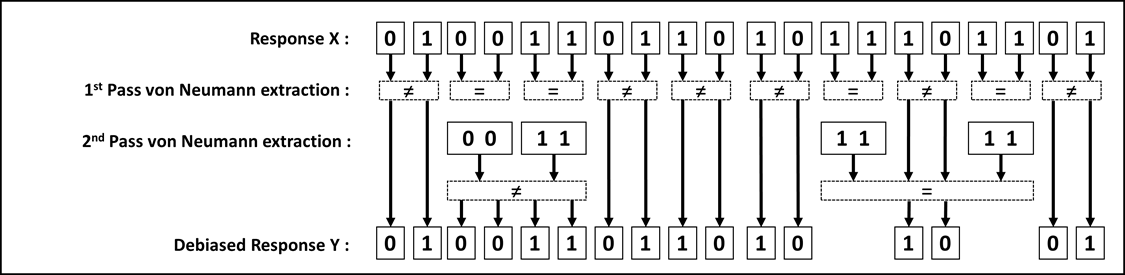
**

**Figure S3. Two-Pass Tuple-Output von Neumann debiasing algorithm process.**

**
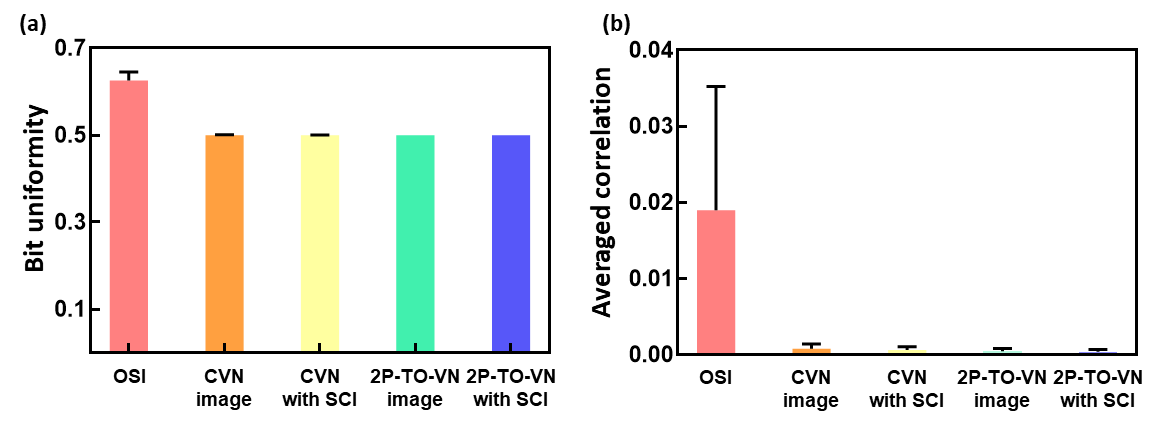
**

**Figure S4. Characterizations comparison of random number matrices of RNGs. (a)** The Von Neumann debiasing process yields a mean value of 0.5 for the unbiased distribution of 0 and 1 states, as evidenced by the bit uniformity calculated from 56 different random bits. **(b)** The average correlation calculated from 56 distinct sets of random bits.

**
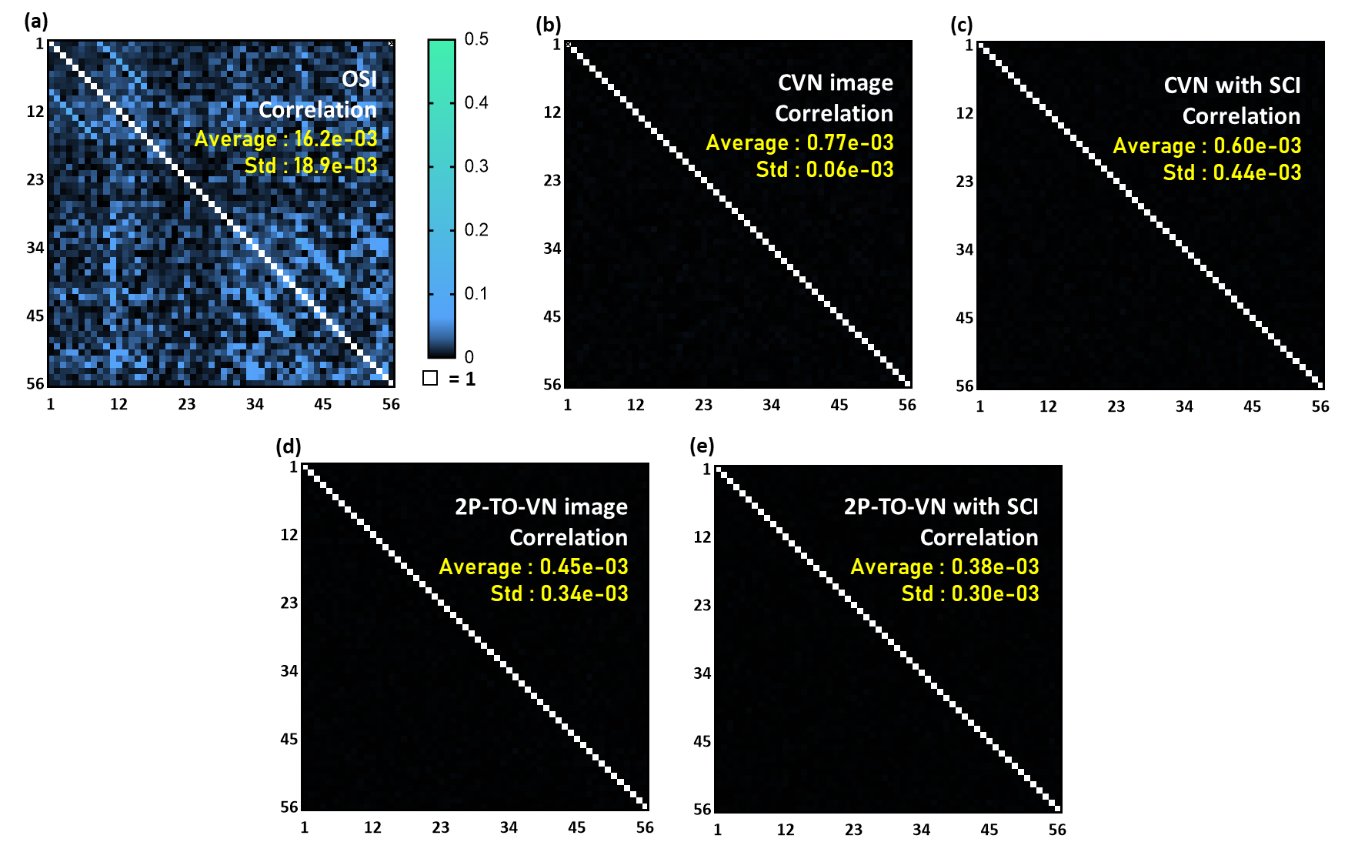
**

**Figure S5. The results of correlation results at the different algorithm types. (a)** Original speckle image, **(b)** CVN image, **(c)** CVN with SCI, **(d)** 2P-TO-VN image, **(e)** 2P-TO-VN with SCI

**
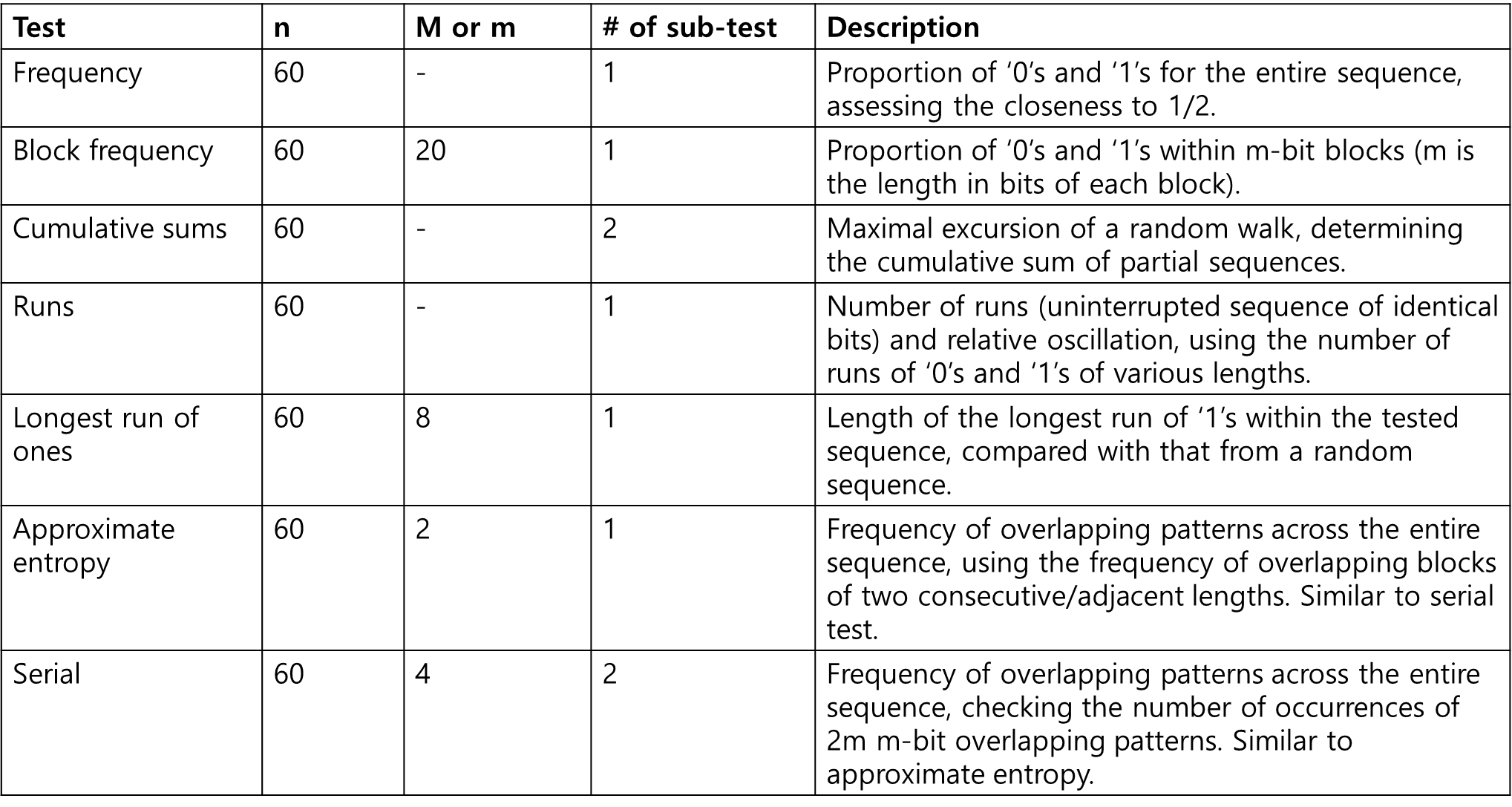
**

**Table S1. Brief characteristic descriptions of the NIST statistical tests.**


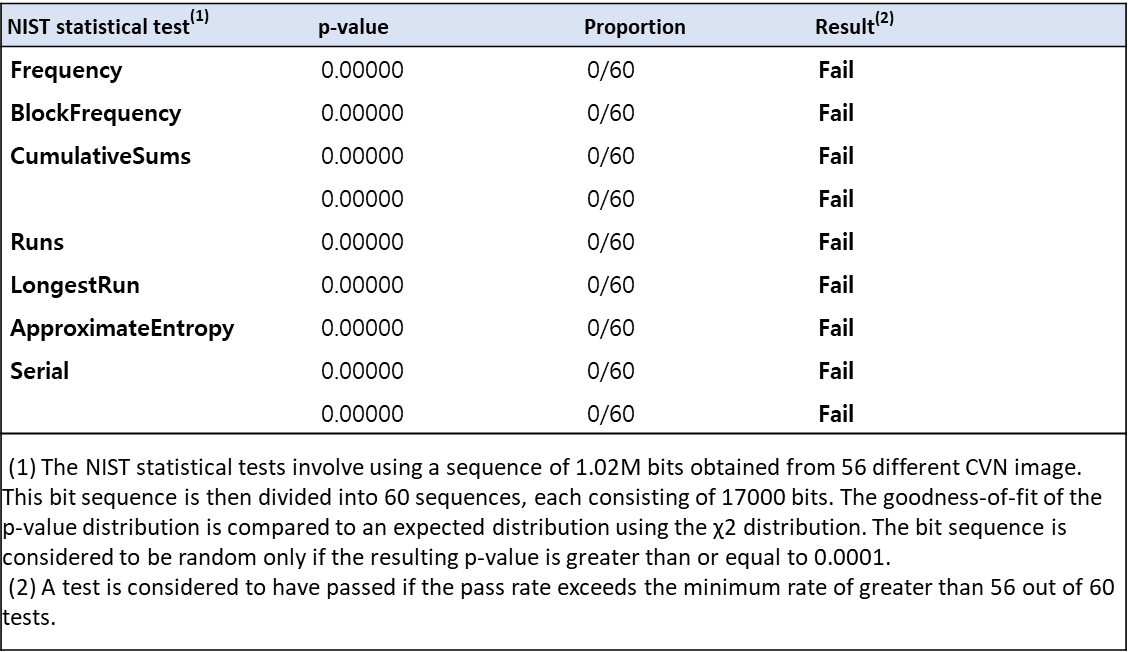


**Table S2. The result of original speckle image by NIST Statistical Randomness Tests for Binary Sequences.**


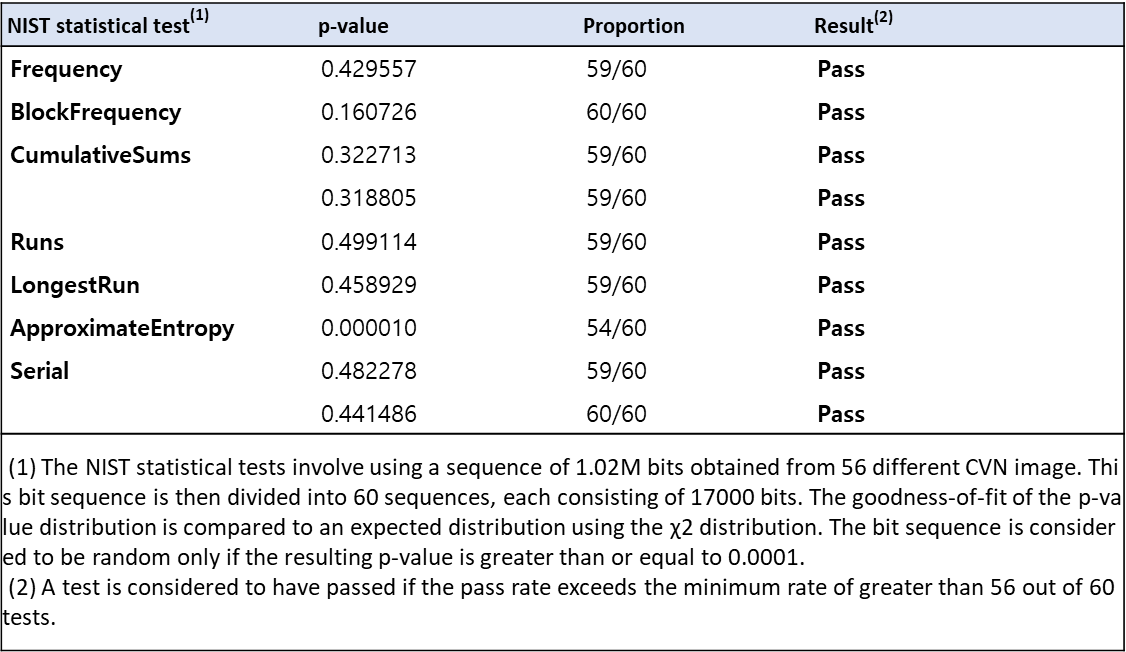


**Table S3. The result of CVN image by NIST Statistical Randomness Tests for Binary Sequences.**


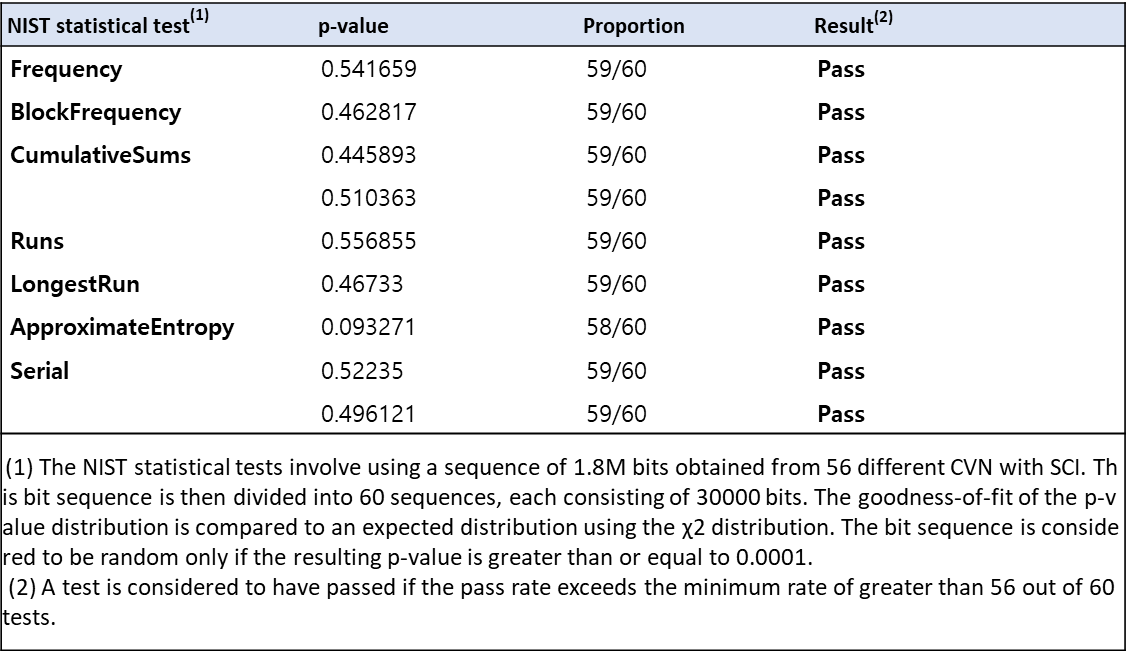


**Table S4. The result of CVN with SCI by NIST Statistical Randomness Tests for Binary Sequences.**
